# Genetic Association between *Matrix Metalloproteinases* Gene Polymorphisms and Risk of Prostate Cancer: A Meta-Analysis

**DOI:** 10.3389/fphys.2017.00975

**Published:** 2017-12-01

**Authors:** Hong Weng, Xian-Tao Zeng, Xing-Huan Wang, Tong-Zu Liu, Da-Lin He

**Affiliations:** ^1^Department of Urology, Zhongnan Hospital of Wuhan University, Wuhan, China; ^2^Center for Evidence-Based and Translational Medicine, Zhongnan Hospital of Wuhan University, Wuhan, China; ^3^Department of Urology, The First Affiliated Hospital of Xi'an Jiaotong University, Xi'an, China

**Keywords:** prostate cancer, matrix metalloproteinases, genetic variation, polymorphism, meta-analysis

## Abstract

**Background and Objective:** Studies suggests that *matrix metalloproteinase* (*MMP*)*-2*-1306 C/T and *MMP-1*-1607 1G/2G polymorphisms affect the risk of prostate cancer. However, the conclusions remain controversial and no pooled evidence of this topic has been published. Therefore, we aimed to perform a meta-analysis to shed some light on the controversial conclusion pertaining to the associations of *MMP-2*-1306 C/T and *MMP-1*-1607 1G/2G polymorphisms with prostate cancer susceptibility.

**Methods:** A thorough literature search was performed up to August, 2016 with the PubMed, EMBASE, CBM, CNKI, and Wanfang databases. Odds ratios (ORs) and corresponding 95% confidence intervals (95% CIs) were calculated to address the correlations between these polymorphisms and risk of prostate cancer.

**Results:** The meta-analysis included six studies (1,921 patients and 1,988 controls) on *MMP-2*-1306 C/T polymorphism and three studies on *MMP-1*-1607 1G/2G polymorphism (438 patients and 394 controls), respectively. The overall results of meta-analysis showed that an elevated risk of the disease was implicated in *MMP-2*-1306 C/T polymorphism under two genetic models (CT vs. CC: OR = 1.78, 95% CI = 1.33–2.38; TT+CT vs. CC: OR = 1.62, 95% CI = 1.24–2.12) and no significant association was observed between *MMP-1*-1607 1G/2G polymorphism and the risk of prostate cancer. The subgroup analysis results of *MMP-2*-1306 C/T polymorphism were similar to the overall results. However, decreased risk of prostate cancer was observed in the Caucasians for *MMP-1*-1607 1G/2G polymorphism.

**Conclusions:** Current meta-analysis indicates that *MMP-2*-1306 C/T polymorphism is associated with elevated risk of prostate cancer, but *MMP-1*-1607 1G/2G polymorphism may inhibit the occurrence of prostate cancer in Caucasians. Further studies are warranted to verify the conclusions.

## Background

Prostate cancer (MIM 176807) is the most common malignancy in males diagnosed in western countries, which is also the second leading cause of death from cancer among men in the US (Torre et al., 2015; Siegel et al., 2016). Prostate cancer was mainly diagnosed with prostate specific antigen (PSA) test, which was commonly applied in the US. With the development of screening technique and the change of lifestyle, the incidence of prostate cancer has been increased in Asian countries, including China (Chen et al., 2016). The disease usually occurs in older men, and the morbidity thereof varies in different ethnic groups which is higher in African Americans for example. Age and racial identity are strong predictors of individual's risk of prostate cancer (Ito, 2014). However, the underlying etiology of prostate cancer is still poorly understood. Numerous studies have demonstrated that genetic background plays a vital role in prostate carcinogenesis and the genetic effect is most important in the context of environment (Haas and Sakr, 1997; Schleutker, 2012; Knipe et al., 2014; Sissung et al., 2014).

Matrix metalloproteinases (MMPs) are a family of proteolytic enzymes that degrade extracellular matrix proteins. MMPs degrade basement membranes and extracellular matrix; the processes are essential for angiogenesis and invasion of carcinoma. More than 20 members in the MMP family have been found in humans (Murphy and Nagase, 2008). The expressions of MMPs are regulated mainly at the level of transcription, and polymorphisms in the promoter region of *MMPs* gene have been considered as transcriptional regulators (Yan and Boyd, 2007). Many studies have examined the associations between *MMP-2*-1306 C/T (rs243865) and *MMP-1*-1607 1G/2G (rs1799750) polymorphisms and risk of prostate cancer (Yaykasli et al., 2014; Adabi et al., 2015; Shajarehpoor Salavati et al., 2017). However, the conclusions are still inconsistent. Furthermore, no meta-analysis has been performed on this topic.

The aim of this meta-analysis was to shed some light on the genetic associations of *MMP-2*-1306 C/T and *MMP-1*-1607 1G/2G gene polymorphisms with prostate cancer susceptibility through incorporating all eligible studies.

## Methods

### Search strategy

A comprehensive systematically literature search was performed up to August, 2016 with the PubMed, EMBASE, CBM, CNKI, and Wanfang databases. Combinations of the following keywords and MeSH terms were used: matrix metalloproteinase; MMP; prostate; prostatic; cancer; carcinoma; neoplasm; polymorphism; variation; genetic; allele; mutation. No language restriction of publication status was applied. In addition, references in the recent reviews were identified for any potentially related studies. We performed this meta-analysis according to Preferred Reporting Items for Systematic Reviews and Meta-Analyses (PRISMA) statement in reporting meta-analysis (Moher et al., 2009). The protocol of the meta-analysis was published in the PROSPERO register (registration number: CRD42016046555). Ethnical approval is not required in this meta-analysis.

### Eligibility criteria

Studies were included if they met the following criteria: (1) the case-control study design was performed; (2) patients were diagnosed with prostate cancer and controls were cancer-free; (3) genotype distribution was presented to calculate odds ratios (ORs) with corresponding 95% confidence intervals (95% CIs); (4) study investigated the association between *MMP-2*-1306 C/T and/or *MMP-1*-1607 1G/2G polymorphism(s) and risk of prostate cancer; (5) study subjects were human beings. The exclusion criteria were as following: (1) review articles, meta-analysis or systematic review, and case reports; (2) the genotype distribution was not available; (3) duplicated publications (studies recently published or with more subjects were included).

### Study selection

Titles and abstracts of papers yielded from online databases based on the prespecified search strategy were screened independently by two reviewers (HW and XTZ) to identify studies that met the prespecified eligibility criteria. The same two reviewers (HW and XTZ) independently retrieved and assessed the potentially eligible publications. Any discrepancy between them over the eligibility of particular articles was settled in consultation with a third reviewer (XHW).

### Data extraction

A standardized, pre-specified form was used to extract data from the included papers for pooled analyses. Extracted information was as following: surname of the first author, publication year, region of the study, ethnicity of the participants, sample size, source of control, genotyping method, distribution of gene frequency, minor allele frequency (MAF), allelic gene frequency, Hardy-Weinberg equilibrium (HWE). Two review authors extracted data independently, and discrepancies were resolved through discussing with a third author if necessary. Missing data would be requested from study authors through e-mail address presented in the paper.

### Statistical analysis

We estimated the associations between polymorphisms and risk of prostate cancer under five genetic models (Yan et al., 2014; Zeng et al., 2014a,b; Weng et al., 2015, 2016; Leng et al., 2016; Zhang et al., 2016). The effects were measured using ORs and the corresponding 95% CIs. Heterogeneity between the studies was assessed using both the χ^2^ test and the *I*^2^ statistic. The *I*^2^ value greater than 50% and/or *P*-value less than 0.1 were indicative of substantial heterogeneity. Sensitivity analysis was conducted based on HWE (Thakkinstian et al., 2005). Besides, stratified meta-analyses by ethnicity and source of control were undertaken for specific relationships. Underlying publication bias was evaluated using Egger's line regression test. All the statistical analyses were performed using Stata 12.0 software. The significance level was set at *P* less than 0.05 except for heterogeneity test.

## Results

### Characteristics of included studies

**Figure 1:**
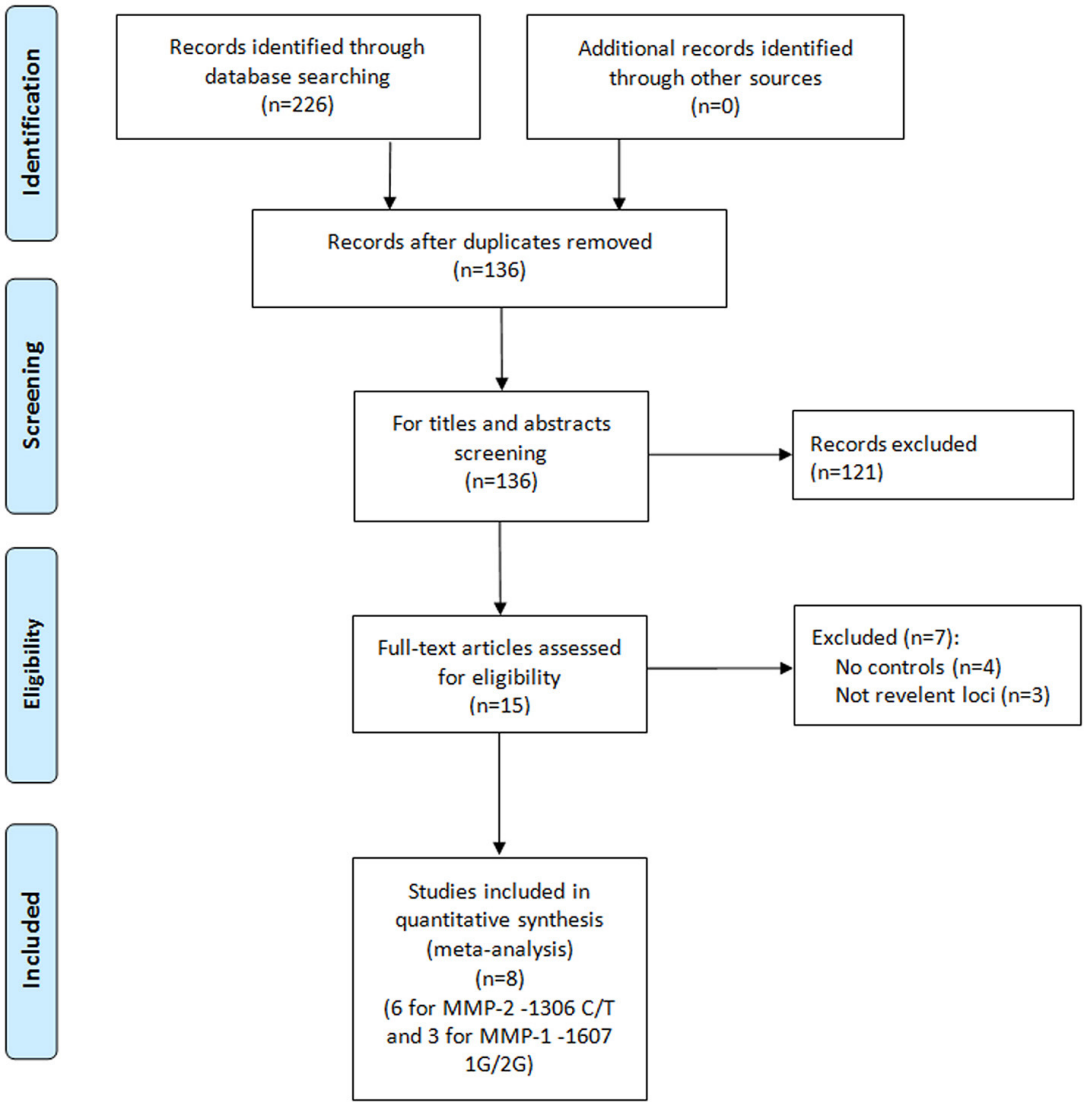
Flow diagram of the search results.

A total of eight papers with nine independent studies were included in this meta-analysis (Figure 1), of which six studies (Jacobs et al., 2008; dos Reis et al., 2009; Srivastava et al., 2012; Yaykasli et al., 2014; Adabi et al., 2015; Shajarehpoor Salavati et al., 2017) examined the association between *MMP-2*-1306 C/T polymorphism and risk of prostate cancer and three studies (Albayrak et al., 2007; dos Reis et al., 2009; Tsuchiya et al., 2009) addressed the association between *MMP-1*-1607 1G/2G polymorphism and risk of prostate cancer. The characteristics of all included studies in the meta-analysis are presented in Table 1. For *MMP-2*-1306 C/T polymorphism, two studies (Adabi et al., 2015; Shajarehpoor Salavati et al., 2017) were conducted in Iran, and only one study was respectively performed in USA (Jacobs et al., 2008), Brazil (dos Reis et al., 2009), Turkey (Yaykasli et al., 2014) and India (Srivastava et al., 2012). One study was involved in mixed populations and others were all related to Caucasians. The controls in one study (Adabi et al., 2015) were benign prostate hyperplasia patients and others were healthy participants. The genotype distributions of controls in two studies (dos Reis et al., 2009; Shajarehpoor Salavati et al., 2017) were not in accordance with HWE, and HWE in the controls of one study (Jacobs et al., 2008) was not clear. For *MMP-1*-1607 1G/2G polymorphism, one study was respectively performed in Turkey (Albayrak et al., 2007), Brazil (dos Reis et al., 2009) and Japan (Tsuchiya et al., 2009). Two studies (Albayrak et al., 2007; dos Reis et al., 2009) referred to Caucasians and one study (Tsuchiya et al., 2009) focused on Asians. The control groups in the three studies were all healthy participants. Thereinto, the genotype distribution of the controls in one study (Albayrak et al., 2007) was not consistent with HWE.

**Table 1:** Characteristics of the studies included in the meta-analysis.

**Study**	**Country**	**Ethnicity**	**Numbers Case Control**		**Control source**	**MAF in controls**	**HWE *P-*value**	**Genotyping method**
**MMP-2-1306 C/T**								
Jacobs et al., 2008^*^	USA	Mixed^#^	1,418	1,449	Healthy	0.24	NA	TaqMan
dos Reis et al., 2009	Brazil	Caucasian	100	100	Healthy	0.31	<0.01	TaqMan
Srivastava et al., 2012	India	Caucasian	190	200	Healthy	0.19	0.92	PCR-RFLP
Yaykasli et al., 2014	Turkey	Caucasian	61	46	Healthy	0.04	0.76	PCR
Adabi et al., 2015	Iran	Caucasian	102	139	BPH	0.09	0.88	PCR-RFLP
Shajarehpoor Salavati et al., 2017	Iran	Caucasian	50	54	Healthy	0.18	<0.01	PCR
**MMP-1-1607 1G/2G**								
Albayrak et al., 2007	Turkey	Caucasian	55	43	Healthy	0.80	<0.01	PCR
dos Reis et al., 2009	Brazil	Caucasian	100	100	Healthy	0.72	0.14	TaqMan
Tsuchiya et al., 2009	Japan	Asian	283	251	Healthy	0.67	0.12	ABI PRISM

*BPH benign prostate hyperplasia, HWE Hardy-Weinberg equilibrium*.

* *Only reported the allele gene frequency*.

# *97% were Caucasian*.

### Heterogeneity test

The between-study heterogeneity of five genetic models of *MMP-2*-1306 C/T polymorphism was low (*I*^2^ range: 0–41.3%; *P*-value range: 0.15–0.98; Table 2). Therefore, fixed-effect model was used for pooling the association between *MMP-2*-1306 C/T polymorphism and risk of prostate cancer. No evidence of significant heterogeneity was detected in heterozygote model (1G/2G vs. 1G/1G) of *MMP-1*-1607 1G/2G polymorphism, so fixed-effects model was applied for meta-analysis. Nevertheless, moderate to high between-study heterogeneity was detected in the remaining four genetic models of *MMP-1*-1607 1G/2G polymorphism (Table 3), thus random-effects model was used for meta-analysis.

**Table 2:** Overall and subgroup analyses of MMP-2-1306 C/T polymorphism and prostate cancer susceptibility.

**Genetic model**	**Subgroup**	**No. of studies**	**Meta-analysis**			**Heterogeneity**	
			**Model**	**OR (95% CI)**	***P-*value**	***I*^2^ (%)**	***P*-value**
T vs. C	Overall	6	FEM	1.11 (1.00–1.23)	0.06	38.3	0.15
	HWE (yes)	3	FEM	1.58 (1.19–2.10)	<0.01	0	0.67
	Ethnicity						
	Caucasians	5	FEM	1.36 (1.09–1.70)	<0.01	0	0.42
	Mixed	1	FEM	1.04 (0.92–1.17)	0.5	–	–
	Control source						
	Healthy	5	FEM	1.09 (0.98–1.22)	0.1	41.3	0.15
	BPH	1	FEM	1.54 (0.86–2.74)	0.15	–	–
TT vs. CC^*^	Overall	5	FEM	1.11 (0.66–1.87)	0.69	8.5	0.36
	HWE (yes)	3	FEM	2.06 (0.87–4.88)	0.1	0	0.55
	Control source						
	Healthy	4	FEM	1.14 (0.67–1.93)	0.63	27.8	0.25
	BPH	1	FEM	0.51 (0.02–12.63)	0.68	–	–
CT vs. CC^*^	Overall	5	FEM	1.78 (1.33–2.38)	<0.01	0	0.95
	HWE (yes)	3	FEM	1.66 (1.18-2.33)	<0.01	0	0.95
	Control source						
	Healthy	4	FEM	1.78 (1.28–2.46)	<0.01	0	0.86
	BPH	1	FEM	1.79 (0.96–3.36)	0.07	–	–
TT vs. CC+CT^*^	Overall	5	FEM	0.89 (0.54–1.48)	0.67	24.2	0.26
	HWE (yes)	3	FEM	1.75 (0.75–4.11)	0.2	0	0.53
	Control source						
	Healthy	4	FEM	0.91 (0.55–1.52)	0.73	41.3	0.16
	BPH	1	FEM	0.45 (0.02–11.12)	0.62	–	–
TT+CT vs. CC^*^	Overall	5	FEM	1.62 (1.24–2.12)	<0.01	0	0.98
	HWE (yes)	3	FEM	1.71 (1.23–2.38)	<0.01	0	0.95
	Control source						
	Healthy	4	FEM	1.60 (1.19–2.16)	<0.01	0	0.95
	BPH	1	FEM	1.72 (0.92–3.20)	0.09	–	–

*OR, odds ratio; CI, confidence interval; HWE, hardy-weinberg equilibrium; FEM, fixed-effects model; BPH, benign prostate hyperplasia*.

* *Populations in these genetic models were all Caucasians*.

**Table 3:** Overall and subgroup analyses of MMP-1-1607 1G/2G polymorphism and prostate cancer susceptibility.

**Genetic model**	**Subgroup**	**No. of studies**	**Meta-analysis**			**Heterogeneity**	
			**Model**	**OR (95% CI)**	***P-*value**	***I*^2^ (%)**	***P*-value**
2G vs. 1G	Overall	3	REM	0.69 (0.40–1.19)	0.18	80	<0.01
	HWE (yes)	2	REM	0.66 (0.31–1.43)	0.29	90	<0.01
	Ethnicity						
	Caucasians	2	REM	0.54 (0.32–0.90)	0.02	44	0.18
	Asians	1	REM	0.96 (0.75–1.24)	0.77	–	–
2G/2G vs. 1G/1G	Overall	3	REM	0.61 (0.25–1.46)	0.27	71.4	0.03
	HWE (yes)	2	REM	0.53 (0.14–2.02)	0.35	85.6	<0.01
	Ethnicity						
	Caucasians	2	REM	0.43 (0.14–1.33)	0.14	62.2	0.1
	Asians	1	REM	1.01 (0.59–1.72)	0.98	–	–
1G/2G vs. 1G/1G	Overall	3	FEM	1.07 (0.69–1.66)	0.77	0	0.68
	HWE (yes)	2	FEM	1.03 (0.65–1.63)	0.89	0	0.48
	Ethnicity						
	Caucasians	2	FEM	0.93 (0.44–1.96)	0.85	0	0.45
	Asians	1	FEM	1.15 (0.67–1.98)	0.61	–	–
2G/2G vs. 1G/1G+1G/2G	Overall	3	REM	0.58 (0.27–1.22)	0.15	79.7	<0.01
	HWE (yes)	2	REM	0.54 (0.18–1.57)	0.26	89.9	<0.01
	Ethnicity						
	Caucasians	2	REM	0.42 (0.19–0.91)	0.03	52.8	0.15
	Asians	1	REM	0.90 (0.64–1.27)	0.56	–	–
2G/2G+1G/2G vs. 1G/1G	Overall	3	REM	0.84 (0.57–1.24)	0.38	34.3	0.22
	HWE (yes)	2	REM	0.83 (0.55–1.27)	0.4	67.1	0.08
	Ethnicity						
	Caucasians	2	REM	0.58 (0.31–1.09)	0.09	0	0.35
	Asians	1	REM	1.07 (0.64–1.78)	0.79	–	–

*OR, odds ratio; CI, confidence interval; HWE, hardy-weinberg equilibrium; FEM, fixed-effects model; REM, random-effects model*.

### *MMP-2*-1306 C/T polymorphism and risk of prostate cancer

**Figure 2:**
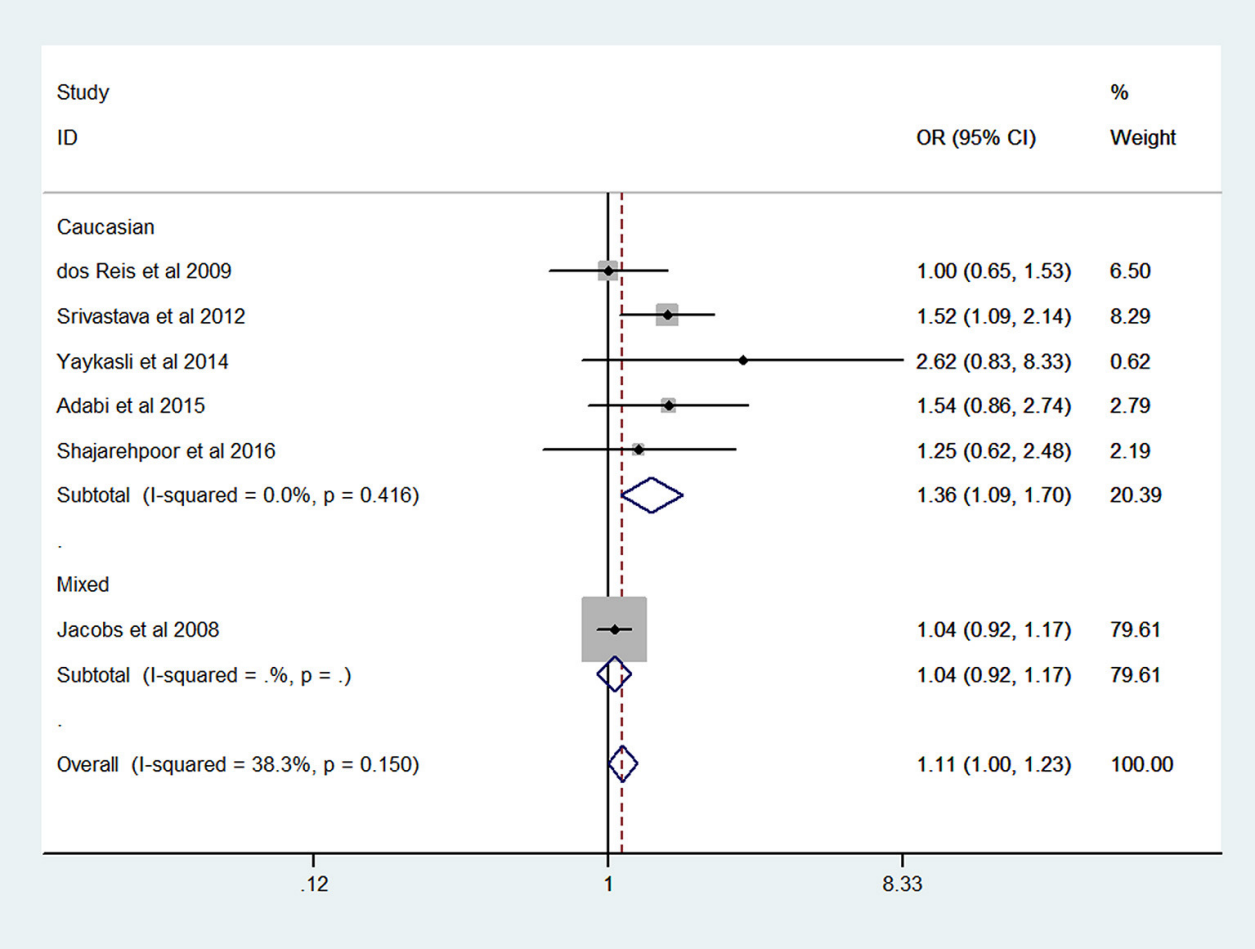
Forest plot of T vs. C genetic model of *MMP-2*-1306 C/T polymorphism.

The results of meta-analysis on *MMP-2*-1306 C/T polymorphism and risk of prostate cancer are presented in Table 2. The overall analysis showed that the *MMP-2*-1306 C/T polymorphism increased the risk of prostate cancer under CT vs. CC model (OR = 1.78, 95% CI = 1.33–2.38) and TT+CT vs. CC model (OR = 1.62, 95% CI = 1.24–2.12). No significant association was observed under the contrasts of T vs. C (OR = 1.11, 95% CI = 1.00–1.23; Figure 2), TT vs. CC (OR = 1.11, 95% CI = 0.66–1.87) and TT vs. CC+CT (OR = 0.89, 95% CI = 0.54–1.48).

Sensitivity analysis was performed by excluding the studies in which the controls were not consistent with HWE. The results of sensitivity analysis were similar to the overall analyses, except for T vs. C model (OR = 1.58, 95% CI = 1.19–2.10). Subgroup analyses showed that an elevated risk of prostate cancer was observed in Caucasians (OR = 1.36, 95% CI = 1.09–1.70; Figure 2) under T vs. C model.

### *MMP-1*-1607 1G/2G polymorphism and risk of prostate cancer

The results of meta-analysis involving *MMP-1*-1607 1G/2G polymorphism and risk of prostate cancer are presented in Table 3. No significant association between *MMP-1*-1607 1G/2G polymorphism and risk of prostate cancer was observed in the overall analyses (2G vs. 1G: OR = 0.69, 95% CI = 0.40–1.19; 2G/2G vs. 1G/1G: OR = 0.61, 95% CI = 0.25–1.46; 1G/2G vs. 1G/1G: OR = 1.07, 95% CI = 0.69–1.66; 2G/2G vs. 1G/1G+1G/2G: OR = 0.58, 95% CI = 0.27–1.22; 2G/2G+1G/2G vs. 1G/1G: OR = 0.84, 95% CI = 0.57–1.24).

The results of sensitivity analysis were similar to the overall analyses by excluding one study in which the controls were not consistent with HWE. Subgroup analysis based on ethnicity showed that *MMP-1*-1607 1G/2G polymorphism decreased the risk of prostate cancer among Caucasians under 2G vs. 1G model (OR = 0.54, 95% CI = 0.32–0.90) and 2G/2G vs. 1G/1G+1G/2G model (OR = 0.42, 95% CI = 0.19–0.91).

### Publication bias

**Figure 3:**
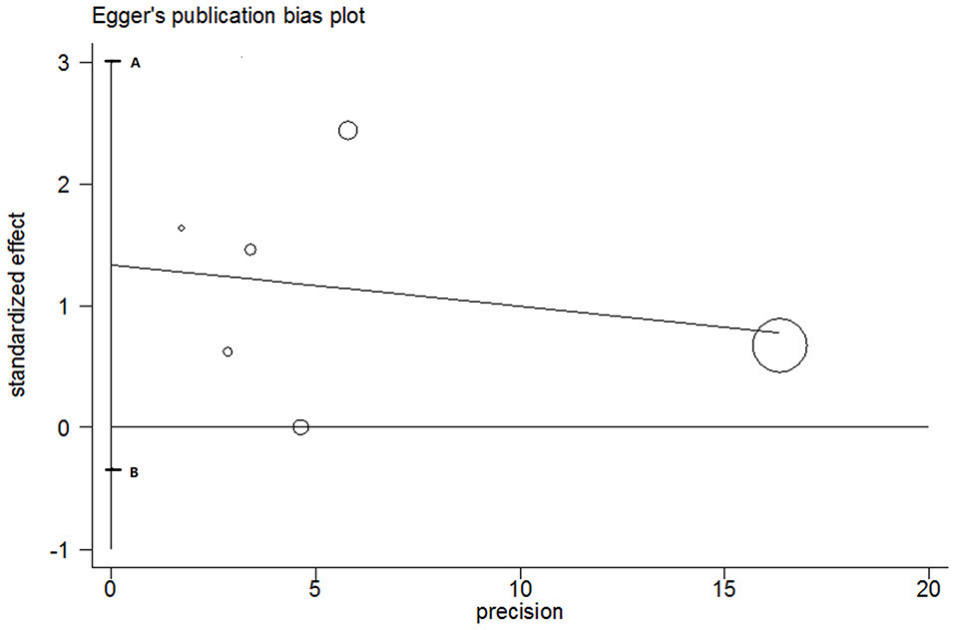
Egger's plot for T vs. C genetic model of *MMP-2*-1306 C/T polymorphism. The segment AB including the zero indicated that no publication bias was existed.

The publication bias was detected using Egger's line regression test for T vs. C model of *MMP-2*-1306 C/T polymorphism. The Egger's publication bias plot (Figure 3) indicated that no obvious publication bias was existed. Egger's test also confirmed the evidence (*P* = 0.09). Due to limited quantity of included studies on *MMP-1*-1607 1G/2G polymorphism, we did not test the publication bias for this polymorphism.

## Discussion

The present meta-analysis, for the first time, comprehensively evaluated the associations between *MMP-2*-1306 C/T and *MMP-1*-1607 1G/2G polymorphisms and risk of prostate cancer. In this meta-analysis, we identified six studies for *MMP-2*-1306 C/T polymorphism and three studies for *MMP-1*-1607 1G/2G polymorphism. The overall analyses showed that the CT and TT+CT carriers presented 1.78- and 1.62-fold higher risk of prostate cancer than CC carriers, respectively. Sensitivity and subgroup analyses showed similar results to the overall analyses, except allelic genetic model. After excluding studies in which controls were not consistent with HWE, the result tended to be significant in Caucasians under the allelic model. The overall analyses of *MMP-1*-1607 1G/2G polymorphism and risk of prostate cancer showed no statistical significance. However, decreased risk of prostate cancer was observed in the Caucasians for *MMP-1*-1607 1G/2G polymorphism.

The decreased risk of prostate cancer concerning *MMP-1*-1607 1G/2G polymorphism in Caucasians might be spurious results or false positives. The significant differences were observed based on only two studies with limited participants. Therefore, the statistical power was relatively weak. Additionally, mutation is usually to be harmful for human and little variation tends to be beneficial. Hence the results indicated that more studies with large sample size were necessary to be carried out for investigating the association.

The most important advantage of our study is that this is the first study to examine the associationsof*MMP-2*-1306 C/T and *MMP-1*-1607 1G/2G polymorphisms with the risk of prostate cancer using meta-analysis. Previous studies were all original case-control studies, which had limited statistical power. Therefore, the results of our study were more precise than that of previous studies. In addition, there are many other polymorphisms in the *MMP-2, MMP-1* and other *MMPs* genes. Therefore, future studies should take these polymorphisms into consideration and haplotype analyses are required to be performed to give more precise results. The prostate cancer is mainly diagnosed by PSA test in clinical practice, and this test has become increasingly prevalent during the last decade. Therefore, the majority of prostate cancer cases are diagnosed with organ-confined cancer (Wirth et al., 2004). But we could not accurately distinguish the risk of advanced or aggressive prostate cancer, which is a life-threatening disease, with indolent nonaggressive cancers using current clinical parameters. Consequently, if the association between *MMP-2*-1306 C/T polymorphism and the risk of prostate cancer was truly effective, researchers ought to explore the association between the polymorphism and the risk of aggressive prostate cancer, which may be a new predicting biomarker for the life-threatening disease.

Several limitations should also be taken into consideration when interpreting the results of this meta-analysis. First, the quantity and sample size of included studies were relatively small even though we undertook a comprehensive literature search, especially for the association between *MMP-1*-1607 1G/2G polymorphism and risk of prostate cancer. Therefore the statistical power was relatively limited (Zeng et al., 2014a; Weng et al., 2015, 2016; Leng et al., 2016). Second, we could not perform haplotype analyses due to limited data. Many other polymorphisms in the *MMP-2* and *MMP-1* genes and other risk genetic polymorphisms might also impact the level of MMPs, such as rs1477017 and 17301608 in *MMP-2* gene (Jacobs et al., 2008). Third, we could not detect the effect of gene-environment due to limited information in the included studies. It is well acknowledged that prostate cancer is a multifactorial and complicated disease involving both gene and environment factors. Fourth, even though we did not find any evidence of publication bias for *MMP-2*-1306 C/T polymorphism, the publication bias could not be avoided. Furthermore, the most populations included in the meta-analysis were in Caucasian populations, therefore the external validity is limited. Lastly, moderate to high between-study heterogeneity was detected for *MMP-1*-1607 1G/2G polymorphism, which might distort the results. Therefore, the results of association between *MMP-1*-1607 1G/2G polymorphism and risk of prostate cancer should be interpreted with caution.

In summary, our findings suggest that the *MMP-2*-1306 C/T polymorphism is associated with elevated risk of prostate cancer, but *MMP-1*-1607 1G/2G polymorphism may have an inhibitory effect on the risk of prostate cancer in Caucasians. More studies with large sample size are warranted to further verify the conclusions in future.

## Author contributions

HW and X-HW: Conceived and designed the study; HW, X-TZ, and X-HW: Participated in study selection, data extraction; HW, T-ZL, and D-LH: Performed statistical analysis; HW, X-TZ, and X-HW: Were involved in manuscript drafting and revision. All authors approved the final manuscript for submission and publication.

### Conflict of interest statement

The authors declare that the research was conducted in the absence of any commercial or financial relationships that could be construed as a potential conflict of interest.
